# Description of the cascade of care and factors associated with attrition before and after initiating antiretroviral therapy of HIV infected children in a cohort study in India

**DOI:** 10.7717/peerj.304

**Published:** 2014-03-13

**Authors:** Gerardo Alvarez-Uria

**Affiliations:** Department of Infectious Diseases, Rural Development Trust Hospital, Bathalapalli, AP, India

**Keywords:** HIV, Pediatrics, India, Rural, Mortality, Lost to follow-up, Antiretroviral therapy highly active, Viral load

## Abstract

In low- and middle-income countries, the attrition across the continuum of care of HIV infected children is not well known. The aim of this study was to investigate predictors of mortality and loss to follow up (LTFU) in HIV infected children from a cohort study in India and to describe the cascade of care from HIV diagnosis to virological suppression after antiretroviral therapy (ART) initiation. Multivariable analysis was performed using competing risk regression. The cumulative incidence of attrition due to mortality or LTFU after five year of follow-up was 16% from entry into care to ART initiation and 24.9% after ART initiation. Of all children diagnosed with HIV, it was estimated that 91.9% entered into care, 77.2% were retained until ART initiation, 58% stayed in care after ART initiation, and 43.4% achieved virological suppression on ART. Approximately half of the attrition occurred before ART initiation, and the other half after starting ART. Belonging to socially disadvantaged communities and living >90 min from the hospital were associated with a higher risk of attrition. Being >10 years old and having higher 12-month risk of AIDS (calculated using the absolute CD4 lymphocyte count and the age) were associated with an increased risk of mortality. These findings indicate that we should consider placing more emphasis on promoting research and implementing interventions to improve the engagement of HIV infected children in pre-ART care. The results of this study can be used by HIV programmes to design interventions aimed at reducing the attrition across the continuum of care of HIV infected children in India.

## Introduction

By the end of 2011, 99% of the 3.3 million HIV infected children worldwide were living in low- or middle-income countries (UNAIDS, 2012). The mortality of children infected with HIV is high (Newell et al., 2004; Spira et al., 1999), especially during the first two years of life, but antiretroviral therapy (ART) reduces the hazard of mortality by 75% (Edmonds et al., 2011). However, only 28% of children who need ART are receiving it (UNAIDS, 2012).

Eventually, almost all HIV infected children will need to initiate ART. Children who are diagnosed with HIV need to follow several stages before the initiation of ART (Fox, Larson & Rosen, 2012). After the HIV diagnosis is made, they need to engage in HIV medical care (Alvarez-Uria et al., 2013a). Once they enter into care, children are followed up until they meet clinical or immunological criteria to start ART (World Health Organization, 2010). After ART initiation, children should be adherent to treatment in order to achieve virological suppression (World Health Organization, 2013). Ideally, all children infected with HIV should be retained across the continuum of care and remain virologically suppressed after initiating ART. Studies performed in HIV infected adults have demonstrated that the attrition across the continuum of care is high in both developed and developing countries (Alvarez-Uria et al., 2013b; Centers for Disease Control and Prevention, 2011; Fox & Rosen, 2010; Mugglin et al., 2012; Rosen & Fox, 2011). However, data about programme attrition in HIV infected children before and after ART initiation are scarce, especially outside sub-Saharan Africa (KIDS-ART-LINC Collaboration, 2008; Munyagwa et al., 2012; Okomo et al., 2012; Tene et al., 2013).

With 145,000 children living with HIV, India has the highest burden of paediatric HIV in Asia (National AIDS Control Organisation, 2013). Of the 112,385 children registered in the HIV national programme, only 34,367 (30.6%) had started ART by December 2012 (National AIDS Control Organisation, 2013). Factors associated with attrition after engagement in care of HIV infected children in India are not well known. The aim of this study is to investigate predictors of mortality and loss to follow up (LTFU) before and after initiating ART in a cohort of HIV infected children in Anantapur, India. In addition, taking into account the results of a previous study from our cohort describing the proportion of HIV infected children who were LTFU before entry into medical care (Alvarez-Uria et al., 2013a), we estimated the proportion of children diagnosed with HIV who follow all the stages of care up to the achievement of virological suppression after initiation of ART.

## Methods

### Setting and design

The study was performed in Anantapur, a district situated in the South border of Andhra Pradesh, India. Anantapur has a population of approximately four million people, and 72% of them live in rural areas (Office of The Registrar General & Census Commissioner, India, 2011). Rural Development Trust (RDT) is a non-governmental organization that provides medical care to HIV infected people free of cost, including medicines, consultations, and hospital admission charges. In our setting, the HIV epidemic is largely driven by heterosexual transmission and it is characterized by poor socio-economic conditions and high levels of illiteracy (Alvarez-Uria et al., 2012b). Although the vast majority of children acquire HIV perinatally, 8% of female children acquire HIV through sexual contacts and 90% of them are diagnosed after 18 months of age. Nearly half of the children have lost one or both of their parents (Alvarez-Uria et al., 2012b).

The Vicente Ferrer HIV Cohort Study (VFHCS) is an open cohort study of all HIV infected patients who have attended RDT hospitals. The characteristics of the cohort have been described in detail elsewhere (Alvarez-Uria et al., 2012a; Alvarez-Uria et al., 2012b). For this study, we selected children (<15 years old) living in Anantapur and diagnosed with HIV between January 1st 2007 and December 31st 2012 from the VFHCS database. The selection of patients from the database was executed on July 15th 2013 (end of the study period).

During this time, ART was started by clinical criteria (clinical stage 3 or 4 of the World Health Organization [WHO]) or by immunological criteria (CD4 count <1500 cells/µl or <25% in children aged <12 months, CD4 count <750 cells/µl or <20% in children aged 12–35 months, CD4 count <350 cells/µl or <15% in children aged 36–59 months, and CD4 count <350 cells/µl in children aged >59 months) according to the Indian National Guidelines (National AIDS Control Organisation, 2006). Children who were LTFU were actively searched for by phone calls and home visits by outreach workers during the study period and, for those children who had died, relatives were asked for the date and location of death.

### Definitions

The designation of the community of patients was performed by self-identification. Scheduled caste (SC) community is marginalised in the traditional Hindu caste hierarchy and, therefore, suffers social and economic exclusion and disadvantage. Scheduled tribe (ST) community is generally geographically isolated with limited economic and social contact with the rest of the population. Backward castes (BC) form a collection of “intermediate” castes that were considered low in the traditional caste hierarchy, but above scheduled castes (Alvarez-Uria, Midde & Naik, 2012). SC, ST and BC communities are considered socially disadvantaged communities and are supported by positive discrimination schemes operated by the Government of India. Children not belonging to SC, ST or BC communities were included in the other castes (OC) group.

Patients were considered as living near a town when they lived in a mandal (administrative subdivision of districts in Andhra Pradesh; e.g., Anantapur District has 64 mandals) containing a town with a population >100,000 people. For those children whose both parents were alive, parents were asked whether they lived in a rented house or owned their house, as a marker of the economic condition of the caregivers.

CD4 lymphocyte count enumeration was performed every six months (Alvarez-Uria et al., 2012d). In HIV infected children <5 years, the CD4 lymphocyte percentage has generally been preferred for monitoring immune status because of the variability of the CD4 cell count during the first years of life (World Health Organization, 2010). However, an analysis of the HIV Paediatric Prognostic Markers Collaborative Study (HPPMCS) demonstrated that the CD4 percentage provides little or no additional prognostic value compared with the CD4 cell count in children (HIV Paediatric Prognostic Markers Collaborative Study et al., 2010). Therefore, the immune status of children was calculated using the 12-month risk of AIDS used by the HPPMCS, which uses the CD4 cell count and the age of children to calculate the level of immunodeficiency (HIV Paediatric Prognostic Markers Collaborative Study, 2006). A high 12-month risk of AIDS indicates low CD4 cell counts for the age and *vice versa*. Because of the small number of older children included in the HPPMCS, children >12 years old were censored at 12 years to calculate the 12-month AIDS risk (“www.hppmcs.org”).

HIV viral load was performed every six months after ART initiation (Alvarez-Uria et al., 2012c). Virological suppression was defined as having an HIV viral load <400 copies/ml in the last viral load determination.

To facilitate the interpretation of continuous variables (e.g., age and distance to the clinics), these were converted into ordinal variables.

### Statistical analysis and Ethics Statement

Statistical analysis was performed using Stata Statistical Software (Stata Corporation. Release 11. College Station, Texas, USA). Time-to-event methods were used. To investigate predictors of mortality or LTFU before ART initiation, time was measured from enrollment in care to ART initiation or death, whatever occurred first (Fox, Larson & Rosen, 2012). To investigate predictors of mortality or LTFU after ART initiation, time was measured from the date of ART initiation to death. Children who did not die were censored at the last visit date. Children who did not come to the clinics for at least 180 days after their last visit date were considered LTFU (Chi et al., 2011). Children who did not die nor were LTFU were considered as retained in care. After completion of each stage, patients who transferred to other clinics were not included in the analysis of the next stage.

Cox regression models assume that the distribution of censoring times and the time to event distribution are independent of each other (Ingle et al., 2010). This assumption is frequently ignored in studies investigating attrition of HIV programmes (Geng et al., 2013). For example, when studying the cumulative incidence of LTFU before ART initiation, a group of children will be censored at death or at the date of ART initiation. However, children who died or started ART cannot be LTFU. Including these children in standard survival models may lead to an overestimation of the event of interest. Thus, univariate and multivariate analysis and estimation of the cumulative incidence of LTFU and mortality were performed using competing risk proportional hazard models (Fine & Gray, 1999). These models estimate sub-distribution hazard ratios (SHRs), which can be interpreted similarly to hazard ratios estimated by Cox proportional models, but they take into account the hazard of the competing events (Ingle et al., 2010). All predictors included in the univariate analysis were included in the multivariate analysis (Kleinbaum & Klein, 2005; Kleinbaum, Klein & Pryor, 2010). The cumulative incidence of mortality and LTFU were estimated using the “stcompet” command in Stata (Cleves, Gould & Gutierrez, 2008; Coviello & Boggess, 2004). The study was approved by the Ethics Committee of the Rural Development Trust Hospital (Reference number OS/003). Written informed consent was given by patients or caretakers for their information to be stored in the study database and used for research.

## Results

**Figure 1:**
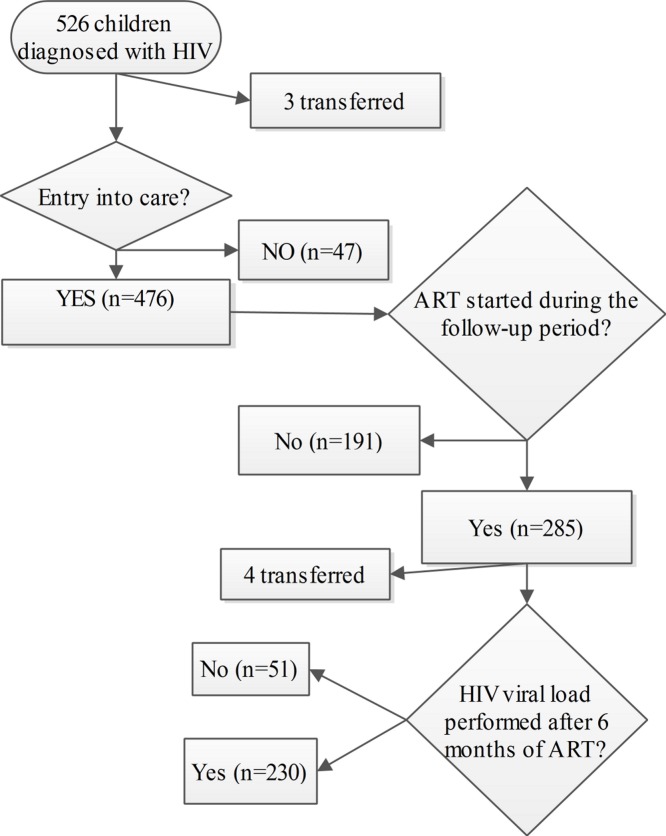
Flowchart of HIV infected children during the continuum of care.

The number of children included in each Stage is summarized in Fig. 1. We identified 526 children diagnosed with HIV infection during the study period. Three children transferred to other centres, nine children died, 38 were LTFU and 476 entered into care.

### From entry into care to ART initiation

Baseline characteristics, univariate and multivariate analysis of factors associated with ART initiation, mortality and LTFU before ART initiation are presented in Table 1. The analysis included 7570 child-months. Of the 476 children who entered into care, 285 started ART, 13 children died and 57 were LTFU. Among children who were LTFU, the median time of follow-up was 5.3 months (interquartile range [IQR], 1.5–19.6) and, the median time from entry into care to ART initiation was 1.9 months (IQR, 0.2–9). Among children who died, the median time from entry into care to death was 7.6 months (IQR, 1.4–16.6). The median age at entry into care was 63.3 months (IQR, 35.7–103.2), and the median 12 months AIDS-risk was 3.8% (IQR, 3.8–8.7; range, 2.6–75). Factors associated with ART initiation were age <2 years or >10 years, earlier calendar year of entry into care, the death of one or both of the parents and having higher 12-month AIDS risk (Table 1). Children who belonged to ST communities were less likely to initiate ART. Belonging to SC communities and having higher 12-month AIDS risk were associated with pre-ART mortality. Children who belonged to ST communities had a higher risk of pre-ART LTFU.

**Table 1:** Baseline characteristics, univariate and multivariable analysis of factors associated with antiretroviral therapy (ART) initiation, mortality and loss to follow up before ART initiation.

		ART initiation		Mortality		Loss to follow up	
	N (%)	SHR	aSHR	SHR	aSHR	SHR	aSHR
**Age (years)**							
0–2	116 (24.47)	2.32^*^ (1.60–3.36)	1.69^*^ (1.11–2.58)	2.54 (0.51–12.74)	1.23 (0.08–17.88)	0.58 (0.25–1.31)	0.77 (0.32–1.85)
3–4	99 (20.89)	1 (Reference)	1 (Reference)	1 (Reference)	1 (Reference)	1 (Reference)	1 (Reference)
5–9	176 (37.13)	1.32 (0.94–1.86)	1.35 (0.95–1.93)	1.07 (0.20–5.72)	1.17 (0.14–9.40)	0.91 (0.47–1.75)	0.87 (0.41–1.81)
>=10	83 (17.51)	2.22^*^ (1.53–3.20)	2.09^*^ (1.44–3.04)	0.57 (0.05–6.28)	0.99 (0.06–15.38)	0.73 (0.31–1.68)	0.71 (0.29–1.73)
**Gender**							
Female	236 (49.58)	0.85 (0.68–1.07)	0.89 (0.70–1.14)	2.33 (0.72–7.59)	2.86 (0.62–13.22)	0.77 (0.46–1.30)	0.74 (0.43–1.28)
Male	240 (50.42)	1 (Reference)	1 (Reference)	1 (Reference)	1 (Reference)	1 (Reference)	1 (Reference)
**Community**							
OC	97 (20.38)	1.22 (0.93–1.61)	1.26 (0.96–1.67)	1.29 (0.23–7.06)	0.89 (0.10–8.18)	0.80 (0.38–1.70)	0.79 (0.36–1.74)
BC	249 (52.31)	1 (Reference)	1 (Reference)	1 (Reference)	1 (Reference)	1 (Reference)	1 (Reference)
SC	104 (21.85)	1.01 (0.74–1.40)	1.06 (0.76–1.48)	3.73^*^ (1.06–13.12)	4.87^*^ (1.21–19.61)	1.15 (0.60–2.23)	1.16 (0.56–2.40)
ST	26 (5.46)	0.43^*^ (0.20–0.90)	0.42^*^ (0.18–0.98)	2.46 (0.28–21.35)	2.76 (0.18–42.03)	2.59^*^ (1.15–5.83)	2.80^*^ (1.17–6.70)
**Living near a town**							
No	297 (62.39)	1 (Reference)	1 (Reference)	1 (Reference)	1 (Reference)	1 (Reference)	1 (Reference)
Yes	179 (37.61)	0.83 (0.65–1.06)	0.85 (0.66–1.11)	0.28 (0.07–1.24)	0.29 (0.06–1.39)	1.50 (0.89–2.52)	1.58 (0.86–2.89)
**Distance to hospital**							
<30 min	132 (27.73)	1 (Reference)	1 (Reference)	1 (Reference)	1 (Reference)	1 (Reference)	1 (Reference)
30–59 min	91 (19.12)	1.25 (0.88–1.77)	1.18 (0.82–1.70)	0.59 (0.12–3.04)	0.41 (0.04–3.87)	1.25 (0.54–2.90)	1.65 (0.67–4.06)
60–90 min	108 (22.69)	1.05 (0.75–1.48)	1.07 (0.75–1.53)	0.74 (0.18–3.12)	0.42 (0.09–2.01)	1.58 (0.74–3.40)	1.55 (0.67–3.59)
>90 min	135 (30.46)	1.21 (0.89–1.66)	1.15 (0.82–1.59)	0.53 (0.13–2.19)	0.41 (0.07–2.55)	1.53 (0.75–3.13)	1.87 (0.90–3.89)
**Year of enrollment**							
2007	81 (17.09)	1 (Reference)	1 (Reference)	1 (Reference)	1 (Reference)	1 (Reference)	1 (Reference)
2008	83 (17.51)	0.79 (0.57–1.09)	0.81 (0.57–1.14)	5.43 (0.74–39.81)	5.69 (0.64–50.47)	0.38 (0.12–1.19)	0.41 (0.13–1.30)
2009	96 (20.25)	0.65^*^ (0.46–0.93)	0.70 (0.48–1.03)	0.91 (0.06–12.90)	1.06 (0.05–23.39)	1.12 (0.49–2.54)	1.20 (0.51–2.81)
2010	93 (19.62)	0.62^*^ (0.43–0.90)	0.73 (0.49–1.08)	1.93 (0.20–18.32)	1.42 (0.12–17.04)	1.46 (0.68–3.15)	1.53 (0.69–3.37)
2011	56 (11.81)	0.61^*^ (0.39–0.96)	0.61^*^ (0.38–0.99)	3.46 (0.37–32.57)	4.46 (0.39–50.94)	1.28 (0.51–3.20)	1.46 (0.57–3.75)
2012	65 (13.71)	0.63^*^ (0.41–0.98)	0.58^*^ (0.35–0.95)	3.26 (0.34–31.19)	3.84 (0.20–74.86)	0.90 (0.33–2.45)	1.12 (0.40–3.14)
**Status of parents**							
Alive, rented house	138 (28.99)	1 (Reference)	1 (Reference)	1 (Reference)	1 (Reference)	1 (Reference)	1 (Reference)
Alive, owned house	113 (23.74)	1.10 (0.78–1.55)	1.25 (0.86–1.81)	0.65 (0.16–2.60)	0.43 (0.06–3.21)	0.51 (0.24–1.06)	0.48 (0.23–1.02)
Father died	115 (24.16)	1.37 (1.00–1.88)	1.62^*^ (1.15–2.27)	0.20 (0.02–1.65)	0.15 (0.01–1.56)	0.57 (0.28–1.14)	0.50 (0.25–1.00)
Mother died	47 (9.87)	1.66^*^ (1.09–2.53)	1.99^*^ (1.26–3.13)	0.49 (0.06–4.02)	0.32 (0.03–3.17)	0.59 (0.23–1.54)	0.68 (0.25–1.87)
Both died	63 (13.24)	1.76^*^ (1.22–2.55)	1.88^*^ (1.25–2.82)	0.69 (0.15–3.23)	0.80 (0.11–5.86)	0.50 (0.21–1.21)	0.48 (0.20–1.19)
**12-month AIDS risk (%)**	3.8 (3.2–8.7)^**^	1.03^*^ (1.02–1.04)	1.03^*^ (1.02–1.04)	1.04^*^ (1.01–1.07)	1.05^*^ (1.01–1.09)	0.98 (0.93–1.02)	0.98 (0.94–1.03)

**Notes.**

* *P*-value <0.05.

** Median (interquartile range).

aSHR adjusted sub-distribution hazard ratio ART antiretroviral therapy BC backward castes CI confidence interval N number OC other castes SHR sub-distribution hazard ratio SC scheduled castes ST scheduled tribes

**Figure 2:**
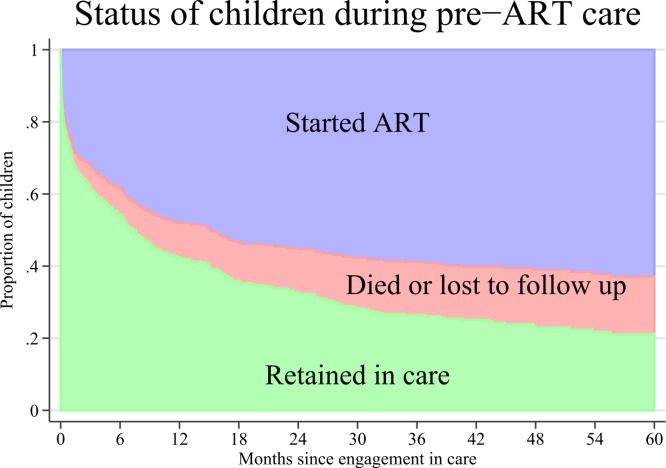
Cumulative incidence of antiretroviral therapy (ART) initiation and attrition (mortality or loss to follow up) after engagement in care of 476 HIV infected children in Anantapur, India.

A stacked graph of the status of HIV infected children since enrollment in care is presented in Fig. 2. The cumulative incidence at 5 years of ART initiation was 63.4% (95% CI [58.3–68.1]) and the cumulative incidence of attrition was 16% (95% CI [12.6–19.7]). The cumulative incidence of mortality and LTFU at 5 years was 2.7% (95% CI [1.5–4.6]) and 13.3% (95% CI [10.2–16.8]), respectively.

### After ART initiation

Of the 285 children who started ART during the study period, four children transferred to other centres and were not included in the analysis. Baseline characteristics, univariate and multivariate analysis of factors associated with mortality and LTFU after ART initiation are presented in Table 2. The analysis included 9856 child-months and, during the study period, 26 children died and 28 were LTFU. Among children who were LTFU, the median time of follow-up was 12.8 months (IQR, 5.3–35.6), and the median time from ART initiation to death was 13.9 months (IQR, 3–22.8). The median age at ART initiation was 68.1 months (IQR, 30.7–109.4), and the median 12 months AIDS-risk was 5.2% (IQR, 3.4–13; range, 2.7–75). Factors associated with mortality after ART initiation were age >10 years and having higher 12-month AIDS risk (Table 2). Children who were >3 months on pre-ART care before the initiation of ART had a lower risk of death. The only factor associated with LTFU after ART initiation was living >90 min from the hospital.

**Table 2:** Baseline characteristics, univariate and multivariate analysis of factors associated with mortality and loss to follow up after antiretroviral therapy initiation.

		Mortality		Loss to follow up	
	N (%)	SHR	aSHR	SHR	aSHR
**Age (years)**					
0–1	45 (16.01)	1 (Reference)	1 (Reference)	1 (Reference)	1 (Reference)
2–4	61 (21.71)	0.45 (0.14–1.43)	1.52 (0.28–8.28)	1.03 (0.34–3.10)	0.98 (0.21–4.56)
5–9	99 (35.23)	0.18^*^ (0.05–0.72)	0.73 (0.11–5.11)	0.94 (0.32–2.77)	1.33 (0.29–5.98)
>=10	76 (27.05)	1.01 (0.38–2.69)	5.30^*^ (1.15–24.48)	0.65 (0.18–2.27)	0.93 (0.16–5.32)
**Gender**					
Female	131 (46.62)	0.83 (0.38–1.80)	0.91 (0.37–2.24)	0.62 (0.29–1.34)	0.66 (0.27–1.60)
Male	150 (53.38)	1 (Reference)	1 (Reference)	1 (Reference)	1 (Reference)
**Community**					
OC	67 (23.84)	0.99 (0.38–2.62)	0.67 (0.23–1.97)	0.65 (0.24–1.75)	0.82 (0.29–2.37)
BC	149 (53.02)	1 (Reference)	1 (Reference)	1 (Reference)	1 (Reference)
SC	57 (20.28)	1.46 (0.57–3.72)	0.85 (0.21–3.49)	1.18 (0.49–2.86)	1.13 (0.32–4.05)
ST	8 (2.85)	1.27 (0.18–8.76)	0.67 (0.12–3.85)	1.00 (0.12–8.36)	1.07 (0.09–13.25)
**Living near a town**					
No	181 (64.41)	1 (Reference)	1 (Reference)	1 (Reference)	1 (Reference)
Yes	100 (35.59)	1.24 (0.56–2.73)	1.09 (0.37–3.20)	1.07 (0.50–2.30)	1.52 (0.61–3.77)
**Distance to hospital**					
<30 min	72 (25.62)	1 (Reference)	1 (Reference)	1 (Reference)	1 (Reference)
30–59 min	55 (19.57)	1.93 (0.68–5.46)	2.55 (0.71–9.14)	0.75 (0.18–3.17)	1.04 (0.22–4.82)
60–90 min	62 (22.06)	1.23 (0.39–3.83)	0.84 (0.19–3.79)	0.51 (0.10–2.61)	0.71 (0.12–4.27)
>90 min	92 (32.74)	0.64 (0.19–2.13)	0.82 (0.20–3.26)	3.17^*^ (1.21–8.30)	5.07^*^ (1.80–14.25)
**Year of ART**					
2007	36 (12.81)	0.93 (0.08–11.20)	0.62 (0.03–12.75)	0.31 (0.08–1.17)	0.38 (0.07–2.07)
2008	44 (15.66)	3.53 (0.41–30.52)	5.04 (0.43–58.87)	0.42 (0.11–1.64)	0.62 (0.12–3.17)
2009	54 (19.22)	2.26 (0.25–20.47)	2.64 (0.19–36.41)	0.61 (0.17–2.13)	0.77 (0.14–4.31)
2010	59 (21)	2.59 (0.28–23.54)	2.90 (0.18–46.20)	0.47 (0.13–1.69)	0.65 (0.14–3.12)
2011	39 (13.88)	3.17 (0.32–31.12)	2.60 (0.13–51.85)	0.33 (0.06–1.81)	0.40 (0.05–3.08)
2012	49 (17.44)	1 (Reference)	1 (Reference)	1 (Reference)	1 (Reference)
**Status of parents**					
Alive, rented house	73 (25.98)	1 (Reference)	1 (Reference)	1 (Reference)	1 (Reference)
Alive, owned house	60 (21.35)	1.49 (0.50–4.43)	0.89 (0.23–3.47)	2.27 (0.86–5.96)	2.38 (0.87–6.52)
Father died	73 (25.98)	0.83 (0.25–2.76)	0.62 (0.11–3.37)	1.03 (0.33–3.18)	1.13 (0.32–3.96)
Mother died	29 (10.32)	0.39 (0.05–3.19)	0.50 (0.06–4.53)	0.41 (0.05–3.38)	0.34 (0.04–2.89)
Both died	46 (16.37)	1.81 (0.61–5.39)	1.72 (0.34–8.76)	1.29 (0.40–4.16)	0.83 (0.20–3.35)
**Pre–ART care**					
<3 months	152 (54.48)	1 (Reference)	1 (Reference)	1 (Reference)	1 (Reference)
>3 months	127 (45.52)	0.42 (0.17–1.07)	0.36^*^ (0.13–0.96)	0.44 (0.18–1.04)	0.42 (0.15–1.21)
**12-month AIDS risk (%)**	4.9 (3.4–12.5)^**^	1.04^*^ (1.02–1.06)	1.04^*^ (1.01–1.08)	1.00 (0.98–1.03)	1.00 (0.97–1.04)

**Notes.**

* *P*-value <0.05

** median (interquartile range).

aSHR adjusted sub-distribution hazard ratio ART antiretroviral therapy BC backward castes CI confidence interval N number OC other castes SHR sub-distribution hazard ratio SC scheduled castes ST scheduled tribes

**Figure 3:**
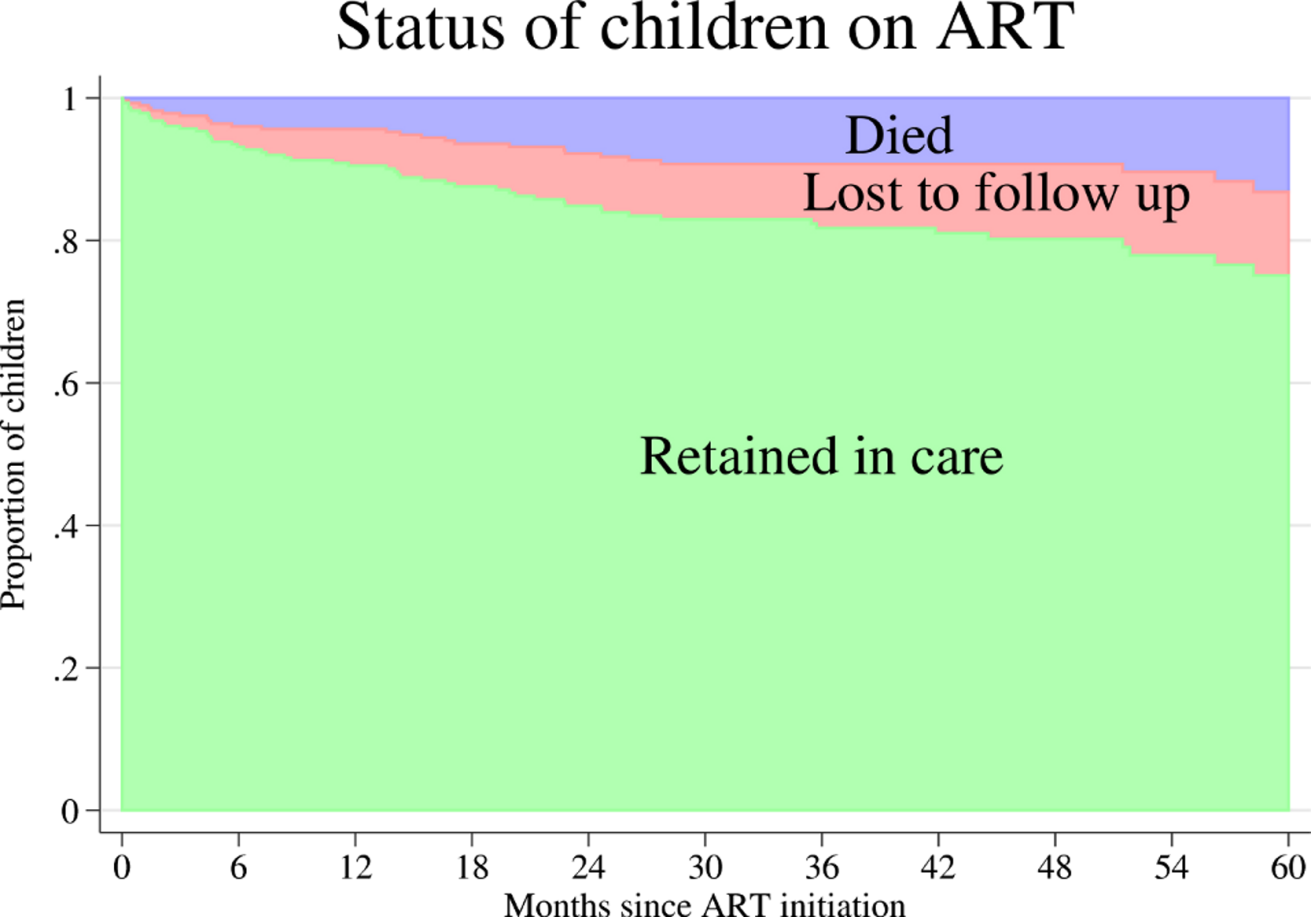
Cumulative incidence of mortality and loss to follow up after antiretroviral therapy (ART) initiation of 281 HIV infected children in Anantapur, India.

A stacked graph of the status of HIV infected children since ART initiation is presented in Fig. 3. The cumulative incidence of attrition (mortality and LTFU) was 24.9% (95% CI [18.7–32.7]) at 5 years. The cumulative incidence of mortality and LTFU at 5 years was 13.2% (95% CI [8.2–19.3]) and 11.7% (95% CI [7.5–16.9]), respectively.

Of the 252 children having >6 months of follow-up after ART initiation, viral load was available in 230 (91.3%) children, and 167 (72.6%) achieved virological suppression in the last viral load determination. The median time from ART initiation to the last viral load determination was 31 months (IQR, 17.6–63.2). The proportion of children who had virological suppression was higher in those who stayed in care (152/203, 74.9%) than in those who died (5/13, 38.5%) or were LTFU (10/14, 71.4%) (*P*-value = 0.017).

### From entry into care to the end of the study period (pre-ART and ART phases combined)

**Figure 4:**
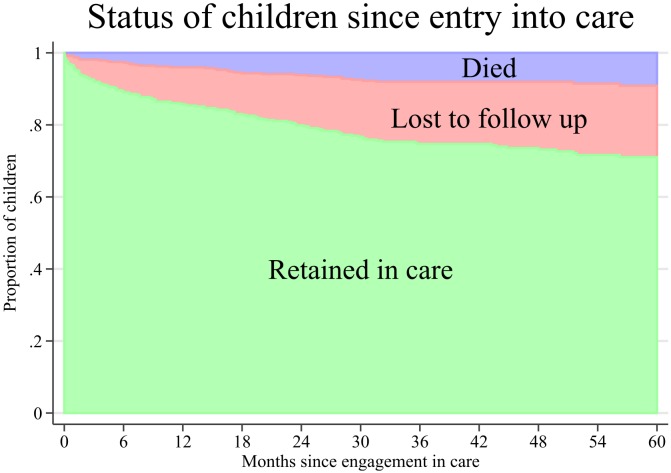
Cumulative incidence of mortality and loss to follow up after engagement in care of 476 HIV infected children in Anantapur, India.

In Fig. 4, we combined pre-ART and ART care to describe the cumulative incidence of attrition since entry into care. The cumulative incidence of attrition (mortality and LTFU) was 29% (95% CI [24.6–33.9]) at 5 years. The cumulative incidence of mortality and LTFU at 5 years was 9.1% (95% CI [6.5–12.4]) and 19.8% (95% CI [16–24]), respectively. Patients transferred to other centres were censored at their last visit date and were not considered as LTFU. Univariate and multivariate analysis of factors associated with mortality and LTFU since entry into care are presented in Table 3. The analysis included 17484 child-months and, during the study period, 39 children died and 85 were LTFU. The majority of deaths (21/39, 53.8%) occurred during a hospital admission in our centre, five occurred in other health-care facility (12.8%) and the rest (13/39, 33.3%) at home. Among children who were LTFU, the median time of follow-up was 8.9 months (IQR, 2–23.5), and the median time from entry into care to death was 14.2 months (IQR, 3.5–26.2). Factors associated with mortality after entry into care were age >10 years, belonging to SC communities and having higher 12-month AIDS risk. Belonging to ST communities and living >90 min from the hospital were associated with LTFU.

**Table 3:** Univariate and multivariate analysis of factors associated with mortality and loss to follow up after engagement in care of 476 HIV-infected children in Anantapur, India.

	Mortality		Loss to follow up	
	SHR	aSHR	SHR	aSHR
**Age (years)**				
0–2	7.23^*^ (1.64–31.91)	1.95 (0.29–12.92)	0.74 (0.40–1.39)	0.83 (0.42–1.65)
3–4	1 (Reference)	1 (Reference)	1 (Reference)	1 (Reference)
5–9	2.23 (0.47–10.57)	1.66 (0.32–8.53)	0.79 (0.46–1.37)	0.77 (0.42–1.42)
> =10	7.02^*^ (1.55–31.86)	7.26^*^ (1.46–36.04)	0.80 (0.41–1.57)	0.80 (0.39–1.64)
**Gender**				
Female	1.08 (0.58–2.02)	1.44 (0.69–3.03)	0.69 (0.45–1.06)	0.66 (0.42–1.04)
Male	1 (Reference)	1 (Reference)	1 (Reference)	1 (Reference)
**Community**				
OC	1.21 (0.52–2.83)	1.00 (0.38–2.61)	0.76 (0.42–1.38)	0.78 (0.43–1.43)
BC	1 (Reference)	1 (Reference)	1 (Reference)	1 (Reference)
SC	2.01 (0.96–4.20)	2.65^*^ (1.08–6.49)	1.12 (0.66–1.91)	1.24 (0.70–2.20)
ST	1.14 (0.27–4.79)	1.52 (0.39–5.95)	1.85 (0.87–3.95)	2.34^*^ (1.00–5.44)
**Living near a town**				
No	1 (Reference)	1 (Reference)	1 (Reference)	1 (Reference)
Yes	0.72 (0.37–1.43)	0.78 (0.36–1.67)	1.30 (0.84–1.99)	1.52 (0.95–2.44)
**Distance to hospital**				
<30 min	1 (Reference)	1 (Reference)	1 (Reference)	1 (Reference)
30–59 min	1.46 (0.63–3.35)	1.30 (0.49–3.46)	1.13 (0.54–2.35)	1.35 (0.62–2.94)
60–90 min	1.03 (0.43–2.49)	0.82 (0.31–2.16)	1.31 (0.67–2.59)	1.32 (0.64–2.70)
>90 min	0.65 (0.26–1.62)	0.67 (0.25–1.83)	2.15^*^ (1.21–3.79)	2.48^*^ (1.38–4.45)
**Year of enrollment**				
2007	1 (Reference)	1 (Reference)	1 (Reference)	1 (Reference)
2008	4.46^*^ (1.56–12.78)	8.03^*^ (2.85–22.62)	0.63 (0.28–1.41)	0.69 (0.30–1.59)
2009	2.65 (0.90–7.80)	3.95^*^ (1.18–13.20)	1.38 (0.72–2.65)	1.55 (0.78–3.08)
2010	1.74 (0.52–5.87)	2.67 (0.72–9.89)	1.69 (0.89–3.20)	1.82 (0.94–3.53)
2011	4.39^*^ (1.36–14.22)	5.35^*^ (1.73–16.51)	1.37 (0.61–3.08)	1.46 (0.64–3.36)
2012	1.72 (0.34–8.83)	1.05 (0.09–12.74)	1.89 (0.84–4.26)	2.01 (0.85–4.78)
**Status of parents**				
Alive, rented house	1 (Reference)	1 (Reference)	1 (Reference)	1 (Reference)
Alive, owned house	1.12 (0.48–2.63)	1.18 (0.39–3.53)	0.87 (0.50–1.53)	0.83 (0.47–1.47)
Father died	0.60 (0.22–1.60)	0.58 (0.16–2.09)	0.72 (0.40–1.29)	0.66 (0.36–1.20)
Mother died	0.50 (0.11–2.21)	0.51 (0.10–2.49)	0.59 (0.25–1.42)	0.64 (0.26–1.58)
Both died	1.62 (0.69–3.79)	1.96 (0.64–6.02)	0.76 (0.38–1.49)	0.71 (0.34–1.49)
**12–month AIDS risk (%)**	1.05^*^ (1.03–1.07)	1.07^*^ (1.04–1.10)	1.00 (0.98–1.02)	1.00 (0.98–1.02)

**Notes.**

* *P*-value <0.05.

** median (interquartile range).

aSHR adjusted sub-distribution hazard ratio ART antiretroviral therapy BC backward castes CI confidence interval N number OC other castes SHR sub-distribution hazard ratio SC scheduled castes ST scheduled tribes

A summary of the cumulative incidences of ART initiation and pre-ART, ART and overall attrition is presented in Table 4. While the attrition in pre-ART stages was mainly due to LTFU, after ART initiation, the cumulative incidence of mortality was similar to the cumulative incidence of LTFU.

**Table 4:** Summary of the cumulative incidence of antiretroviral therapy (ART) initiation, and attrition due to mortality and lost to follow up (LTFU) of HIV infected children from Anantapur, India.

	**3 months**	**6 months**	**1 year**	**2 years**	**3 years**	**4 years**	**5 years**
**ART initiation**	32.8 (28.6–37)	38.7 (34.3–43.1)	48.5 (43.9–52.9)	55.5 (50.9–59.9)	59.2 (54.6–63.7)	61.5 (56.7–65.9)	63.4 (58.3–68.1)
**Pre–ART attrition**	5.7 (3.8–8)	7.4 (5.2–10)	9.5 (7.1–12.3)	12.1 (9.3–15.2)	14.7 (11.6–18.2)	15.5 (12.3–19.1)	16 (12.6–19.7)
Mortality	0.8 (0.3–2)	0.8 (0.3–2)	1.7 (0.8–3.2)	2.2 (1.1–3.8)	2.7 (1.5–4.6)	2.7 (1.5–4.6)	2.7 (1.5–4.6)
LTFU	4.8 (3.2–7)	6.5 (4.5–9)	7.8 (5.6–10.4)	9.9 (7.4–12.9)	12 (9.2–15.3)	12.8 (9.8–16.2)	13.3 (10.2–16.8)
**ART attrition**	4.3 (2.5–7.5)	6.9 (4.5–10.6)	9.5 (6.6–13.7)	15.1 (11.3–20.2)	18.3 (13.9–23.8)	19.8 (15.1–25.8)	24.9 (18.7–32.7)
Mortality	2.5 (1.1–4.9)	4 (2–6.8)	4.4 (2.4–7.3)	7.8 (4.9–11.5)	9.3 (6–13.3)	9.3 (6–13.3)	13.2 (8.2–19.3)
LTFU	1.8 (0.7–3.9)	2.9 (1.4–5.4)	5.2 (3–8.3)	7.3 (4.6–10.9)	9 (5.8–13.1)	10.6 (6.8–15.2)	11.7 (7.5–16.9)
**Overall attrition**	7.6 (5.5–10.4)	10.8 (8.3–13.9)	14.2 (11.4–17.7)	20.1 (16.7–24.1)	25.3 (21.4–29.7)	26.4 (22.4–31)	29 (24.6–33.9)
Mortality	1.9 (0.9–3.4)	2.5 (1.4–4.2)	4 (2.5–6.1)	6.2 (4.2–8.6)	8 (5.7–10.8)	8 (5.7–10.8)	9.1 (6.5–12.4)
LTFU	5.7 (3.8–8)	8.2 (6–10.9)	10.2 (7.6–13.1)	14 (11–17.3)	17.3 (13.9–21)	18.4 (14.8–22.3)	19.8 (16–24)

### Description of the cascade of care

**Figure 5:**
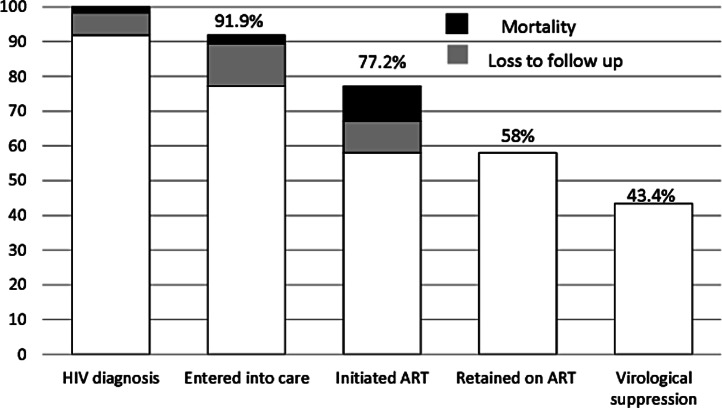
Cumulative retention of children at each stage of care after HIV diagnosis.

Taking into account the number of children at each stage of care and the attrition before entering into care (8.1% after six years of follow-up) (Alvarez-Uria et al., 2013a), from entry into care to ART initiation (16%), and after ART initiation (24.9%), and the proportion of children who were retained in care and did not achieve virological suppression (25.1%), we estimated that 43.4% of children who were diagnosed with HIV were retained in care up to the achievement of virological suppression (Fig. 5). Overall, 65.7% of the attrition was due to LTFU and 34.3% was due to mortality; 19.3% of the attrition occurred before entry into care, 35% from entry into care to ART initiation, and 45.7% after ART initiation. The attrition due to LTFU was higher than the one due to mortality before entry into care (79 versus 21%), and from entry into care to ART initiation (83 versus 17%), but not after ART initiation (47 versus 53%).

## Discussion

The results of this study show that fewer than half of the children diagnosed with HIV followed all stages of care from the HIV diagnosis to the achievement of virological suppression after ART initiation. To the best of our knowledge, this is one of the first studies to describe the cascade of care in a cohort of HIV infected children.

Although comparisons among studies are difficult because of different study methodologies, our findings are in accordance with other paediatric studies describing the attrition at particular stages of care. In a systematic review of the retention in care of HIV infected children from resource-limited settings, the attrition before entry into care was 9.7%–22%, and 0.4–60.5% from ART eligibility to ART initiation (Mugglin et al., 2013). In a multiregional collaboration of paediatric cohorts from Asia and Africa, the adjusted risk of death after ART initiation was 4.3–7.4% and the adjusted risk of LTFU was 4.1–21.8% (Leroy et al., 2013). These data highlight the need to improve the retention in care of HIV infected children in low- and middle-income countries.

To date, the majority of HIV research and funding has focused on the reduction of morbidity and mortality of children after ART initiation (Mugglin et al., 2013). However, the results of this study indicate that the attrition in pre-ART stages of care is similar to the attrition after ART initiation, and mostly due to LTFU. HIV programmes should consider placing more emphasis on promoting research and implementing interventions to improve the engagement of HIV infected children in pre-ART care.

Data from developed countries estimate that more than half of HIV infected people are lost across the continuum of care (Mugavero et al., 2013). In a study from our cohort, only 38% of adults diagnosed with HIV started ART and were retained in care (Alvarez-Uria et al., 2013b). The results of the present study suggest that the attrition across the continuum of care in children is lower than the attrition in adults. However, the proportion of adults on ART who achieve virological suppression is approximately 80% (Alvarez-Uria et al., 2013b; Mugavero et al., 2013), which is substantially higher than the proportion of virologically suppressed children observed in this study, suggesting that it is more difficult to achieve virological suppression in children than in adults (Barth et al., 2011; Turkova, Webb & Lyall, 2012).

In this study, we present factors associated with mortality and LTFU that could be used to reduce the attrition of HIV programmes in India. The 12-month AIDS risk, which was calculated using the age and the CD4 lymphocyte count, was strongly associated with mortality, suggesting that this parameter could be useful to calculate the level of immunodeficiency in children from resource-limited settings (HIV Paediatric Prognostic Markers Collaborative Study et al., 2010). Interestingly, children who initiated ART at age >10 years had an increased risk of death independent of their 12-month AIDS risk. Studies from developed countries have demonstrated similar risks of AIDS and death in adults and children aged >5 years (Dunn et al., 2008; HIV Paediatric Prognostic Markers Collaborative Study, 2006), but this has not been confirmed in low- or middle-income countries with higher prevalence of HIV-related diseases such as tuberculosis. With the implementation of the new WHO guidelines (World Health Organization, 2013), the proportion of children who will start ART sooner will increase dramatically, and the results of this study suggest that this could have a beneficial effect on paediatric HIV mortality. However, new studies are needed to clarify the increased risk of death in children >10 years old after ART initiation. Children living >90 min from the hospital had an increased risk of LTFU, which supports the current policy of decentralization of HIV centres by the Indian Government.

Children who started ART within three months of entry into care and those children belonging to socially disadvantaged communities (ST and SC) were at higher risk of attrition. In sub-Saharan Africa, the most common reasons for not taking HIV infected children to the clinics were transport costs, food availability, and time constraints due to work commitment (Wachira et al., 2012), so families belonging to socially disadvantaged communities may require economic support from HIV programmes to improve the retention of the care of their children.

The study has some limitations. We estimated the proportion of children who were not retained in pre-ART care, but not all of them were followed up until starting ART. Therefore, it is possible that longer follow-up periods may result in higher rates of pre-ART attrition. Children LTFU at any stage may not be lost forever, as they may reengage in the future or enroll in other HIV centres. However, patients LTFU are more likely to initiate ART with low CD4 counts or may die before attending other healthcare facilities (Brinkhof, Pujades-Rodriguez & Egger, 2009). The eligibility criteria for starting ART during the study period followed the 2006 WHO Guidelines (National AIDS Control Organisation, 2006). In the 2010 and 2013 WHO guidelines (World Health Organization, 2010; World Health Organization, 2013), the age and the CD4 count threshold for starting ART has been raised considerably, so it is likely that the pre-ART period of care would be shortened and, ideally, children will start ART at higher CD4 counts. New studies in settings where these guidelines are implemented are needed to clarify the effect of the new WHO guidelines on the attrition of HIV programmes.

## Conclusions

This study shows that fewer than half of the children diagnosed with HIV in our setting follow all stages of care up to the achievement of virological suppression after ART initiation. Half of the attrition occurred before ART initiation, suggesting that paediatric HIV programmes in low- and middle-income countries should monitor their performance in the pre-ART stages of care. The 12-month AIDS risk, which is calculated using the CD4 lymphocyte count and the age, was a strong predictor of mortality, suggesting that this parameter could be useful for resource-limited settings. Children aged >10 years and those who started ART within three months of enrollment in care had a higher risk of mortality, which could not be explained by the level of immunodeficiency. Children belonging to socially disadvantaged communities were at higher risk of attrition. The results of this study can help design interventions to reduce the mortality and LTFU of HIV infected children in India.

## Supplemental Information

10.7717/peerj.304/supp-1 Supplemental Information 1 Ethical clearance
