# Supplementary figures and images for: Description of the cascade of care and factors associated with attrition before and after initiating antiretroviral therapy of HIV infected children in a cohort study in India
Source: PeerJ. 2014 Mar 13;2:e304. doi: 10.7717/peerj.304 (PMC3961166; doi:10.7717/peerj.304)

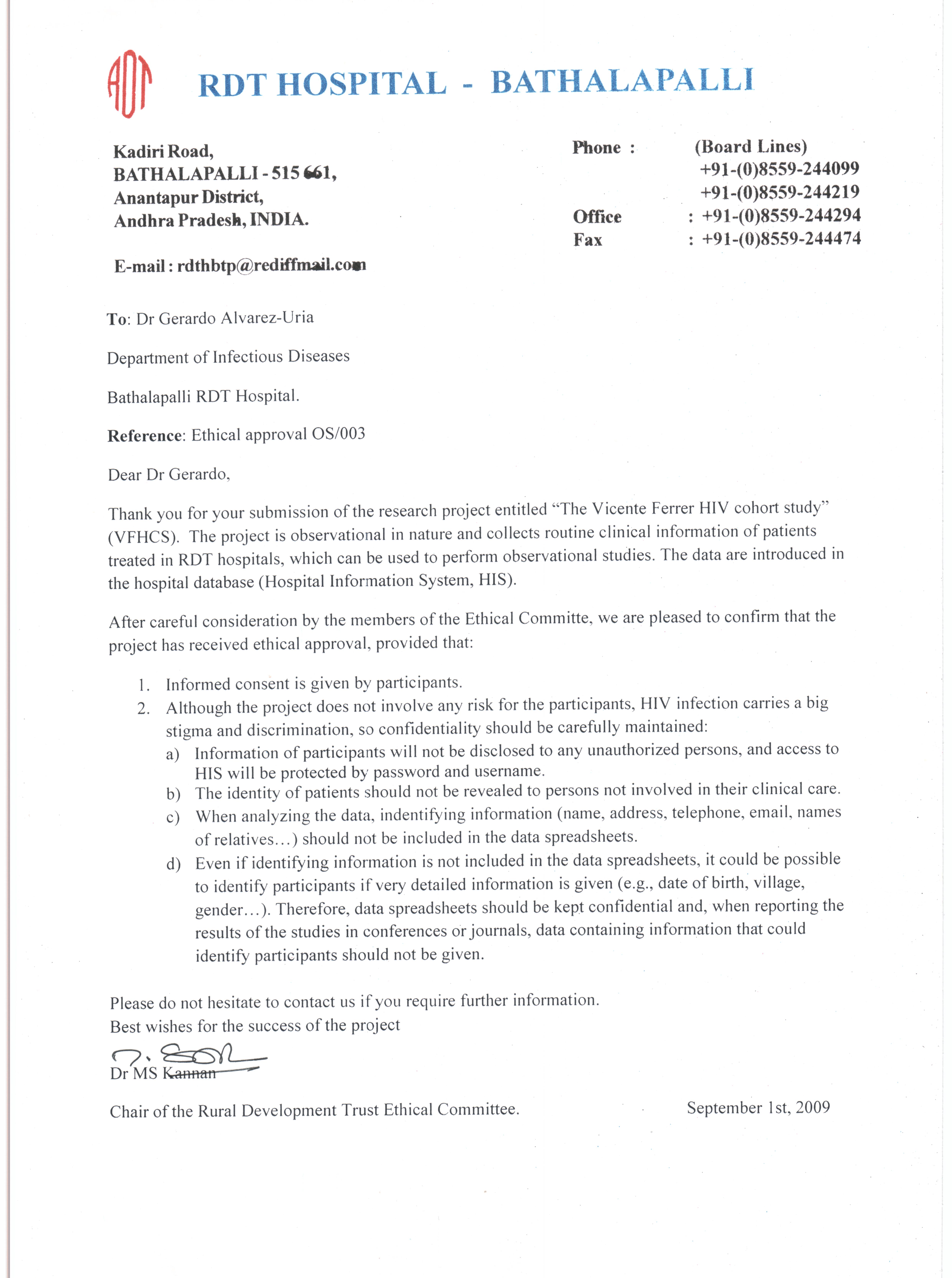

Supplement: Supplemental Information 1 [file peerj-02-304-s001.jpg]
